# Downregulation of LIMK1–ADF/cofilin by DADS inhibits the migration and invasion of colon cancer

**DOI:** 10.1038/srep45624

**Published:** 2017-03-30

**Authors:** Jian Su, Yujuan Zhou, Zhibing Pan, Ling Shi, Jing Yang, Aijun Liao, Qianjin Liao, Qi Su

**Affiliations:** 1 Department of Pathology, The Second Affiliated Hospital, University of South China, Hengyang, Hunan, China; 2 Key Laboratory of Cancer Cellular and Molecular Pathology of Hunan Provincial University, Cancer Research Institute, University of South China, Hengyang, Hunan, China; 3 Key Laboratory of Translational Radiation Oncology, Hunan Province, Hunan Cancer Hospital and The Affiliated Cancer Hospital of Xiangya School of Medicine, Central South University, Changsha, Hunan, China; 4 Department of Gastroenterology, The First Affiliated Hospital, University of South China, Hengyang, Hunan, China

## Abstract

This study aimed to explore whether the downregulation of LIM kinase 1 (LIMK1)-actin depolymerization factor (ADF, also known as destrin)/cofilin by diallyl disulfide (DADS) inhibited the migration and invasion of colon cancer. Previous studies have shown that silencing LIMK1 could significantly enhance the inhibitory effect of DADS on colon cancer cell migration and invasion, suggesting that LIMK1 was a target molecule of DADS, which needed further confirmation. This study reported that LIMK1 and destrin were highly expressed in colon cancer and associated with poor prognosis of patients with colon cancer. Also, the expression of LIMK1 was positively correlated with the expression of destrin. The overexpression of LIMK1 significantly promoted colon cancer cell migration and invasion. DADS obviously inhibited migration and invasion by suppressing the phosphorylation of ADF/cofilin via downregulation of LIMK1 in colon cancer cells. Furthermore, DADS-induced suppression of cell proliferation was enhanced and antagonized by the knockdown and overexpression of LIMK1 *in vitro* and *in vivo*, respectively. Similar results were observed for DADS-induced changes in the expression of vimentin, CD34, Ki-67, and E-cadherin in xenografted tumors. These results indicated that LIMK1 was a potential target molecule for the inhibitory effect of DADS on colon cancer cell migration and invasion.

Colon cancer is one of the most common malignant tumors in humans. It is the third largest cause of cancer death, and its incidence and mortality rates have increased year after year^1^. Currently, it has been recognized that the poor prognosis of patients with advanced colon cancer is mainly related to resistance to chemotherapy, and metastasis occurs in about 50% of patients with colon cancer within 5 years^1^. Thus, exploring the molecular mechanisms underlying colon cancer metastasis may assist in preventing and treating colon cancer.

During the early stages of tumor cell invasion and metastasis, various pseudopodia (such as filopodia, lamellipodia, invadopodia, and podosomes) are formed^2^,^3^. Pseudopodia formation and movement depend on the continuous polymerization and depolymerization of actin that can be affected by several factors. LIM kinase (LIMK) can remodel the cytoskeleton by regulating the activity of actin depolymerization factor (ADF, also known as destrin)/cofilin, which represents a family of actin-binding proteins, thereby playing an important role in the formation of filopodia^4^,^5^. LIMK family includes LIMK1 and LIMK2, which are protein kinases regulating the polymerization of actin and decomposition of microtubules^6^,^7^. The activity of LIMK1 is regulated by upstream molecules such as Rho GTPases, p21-activated kinase, and Rho kinase^6^,^8^. LIMK1 activation can promote the phosphorylation of ADF/cofilin (downstream signaling molecules of LIMK1), result in reducing the depolymerization of ADF/cofilin and regulating actin cytoskeletal reorganization, which further promote tumor cell filopodia formation and metastasis^9^. LIMK1 has been shown to be overexpressed or overactive in a variety of malignant tumors. It is involved in tumor cell proliferation, migration, invasion, and metastasis^10^. Cofilin one of downstream signaling molecules of LIMK1 belongs to the ADF/cofilin family, it overexpressed in the invasive tumour cells and other cells^11^,^12^,^13^. Many studies have indicated that cofilin pathway is essential for cell motility and morphogenesis *in vitro* and *in vivo*, and the cofilin pathway has also been implicated in tumour cell invasion and metastasis^14^,^15^. Interfering with either LIMK1 or ADF/cofilin can inhibit tumor cell proliferation, migration,and invasion, suggesting that LIMK1 may influence the ADF/cofilin activity, which plays an important role in tumor invasion and metastasis^16^,^17^.

Garlic not only is a very popular food but also hasmedicinal value. It has antitumor properties. It can help in the sterilization and prevention of a variety of cardiovascular and cerebrovascular diseases, without any toxic side effects on the body^18^,^19^. Studies have shown that diallyl disulfide (DADS) is the main component of garlic having an anticancer effect on gastric cancer^20^, breast cancer^21^, leukemia^22^, and other tumors. Proteomics studies demonstrated that DADS significantly decreased the expression of LIMK1 and ADF/cofilin^23^. DADS inhibited epithelial–mesenchymal transition (EMT), invasion, and proliferation by downregulating LIMK1 in gastric tumor cells^24^. DADS-induced suppression of cell proliferation was enhanced and antagonized by the knockdown and overexpression of LIMK1 *in vitro* and *in vivo*, respectively^24^. Furthermore, DADS was shown to inhibit colon cancer cell proliferation, migration, and invasion owing to the negative regulation of LIMK1–ADF/cofilin signaling pathway. Silencing the expression of LIMK1 may enhance the inhibitory effect of DADS on colon cancer cell migration and invasion^25^. Thus, it was speculated that LIMK1 might be a key target molecule for the inhibitory effect of DADS on cell migration and invasion of human colon cancer, which needed further confirmation. The present study found that LIMK1 and destrin (also known as ADF) were highly expressed in colon cancer. The overexpression of LIMK1 significantly accelerated the phosphorylation of ADF/cofilin, hence promoting colon cancer cell migration and invasion. DADS inhibited the phosphorylation of ADF/cofilin by downregulating the expression of LIMK1, thereby inhibiting colon cancer migration and invasion. These findings suggested that DADS had a significant anticancer effect, indicating that LIMK1 is a potential target molecule for the inhibitory effect of DADS on tumor cell migration and invasion.

## Materials and Methods

### Cell culture andestablishment of LIMK1 stably overexpressing cell line

Human colon cancer cell line SW480 (a kind gift from the Cancer Research Institute, Xiangya Medical College, Central South University) was maintained in the RPMI1640 complete medium (Gibco, Life Technologies, Vienna, Austria) containing 10% fetal bovine serum in a humidified incubator at 37 °C with 5% CO_2_. SW480 human colon cancer cells were transfected with pIRES2-LIMK1 eukaryotic expression plasmid and pIRES2-enhanced green fluorescent protein empty vector using Lipofectamine 2000 to generate an LIMK1 stably overexpressing colon cancer cell line. G418 was used to screen positive cell clones. Quantitative reverse transcription–polymerase chain reaction (qRT-PCR) and Western blot were used to confirm the establishment of LIMK1 stably overexpressing cell line. The stable colon cancer cell line with silencing of the expression of LIMK1(LIMK1-miR/SW480) and empty vector (miR/SW480) was established in a previous study^20^, and the sequences of DNA oligomers inserted into pcDNA6.2-GW/EmGFPmiR (constructed by Invitrogen Corporation, NY, USA) were as follows: sense, 5′-TGCTGATGGAGTGGAGGTAGGCCATCGTTTTGGCCACTGACTGACGATGGCCTCTCCACTCCAT -3′ and antisense, 5′-CCTGATGGAGTGGAGAGGCCATCGTCAGTCAGTGGCCAAAACGATGGCCTACCTCCACTCCATC-3′.

### Clinical data and chip production

All experimental procedures wereapproved by the ethics committee of the University of South China, and informed consent was obtained from all patients. The procedures followed for collection and use of tissues were in accordance with the ethical standards formulated in the Declaration of Helsinki. A total of 87 surgically resected colon cancer specimens and 34 normal tissues adjacent to colon cancer were collected from 2007 to 2011 at the Affiliated Hospital of University of South China. The clinical and pathological features are shown in Table 1. The average follow-up was 47.45 months (6–96 months). All patients did not receive radiotherapy and chemotherapy.

### Tissue microarray

Leica paraffin (Leica Histowax, Leica Biosystems, Germany) and beeswax (Hualing, Shanghai, China) were mixed in aratio of 7:3 to prepare a recipient paraffin block of size 2.2 × 3.5 × 1 cm^3^ and design tissue arrays of 5 × 10 points. A tissue microarray instrument (Beecher Instruments Inc., WI, USA) was used to punch and produce a blank wax block with holes. The tissue paraffin block was placed in a water bath at 39–42 °C for 3 min, and a 2-mm needle (Beecher Instruments Inc., WI, USA) was used to select lesions. Then, tissues of the size 2 × 3 mm^2^ were removed one by one and placed into the holes of the blank wax block, resulting in the arrangement of a tissue microarray.

### Immunohistochemical staining

The paraffin sections were deparaffinized, followed by hydration, using an immunohistochemistry kit (Maixin Biotech. Co., Fuzhou, China) according to the manufacturer instructions. LIMK1, destrin, E-cadherin, vimentin, Ki-67, and CD34 antibodies (Abcam, Cambridge, UK) were incubated overnight at 4 °C, and normal rabbit immunoglobulin G was considered as a negative control. The specimens were visualized with 3,3′-diaminobenzidine and counterstained with hematoxylin. The staining was brown or tan and located in the cytoplasm or nucleus. The scores according to the degree of positive staining and the percentage of stained cells were as follows: 0, no coloring; 1, light brown; 2, dark brown; 0, staining cells < 5%; 1, staining cells between 5% and 25%; 2, staining cells between 26% and 50%; 3, staining cells > 50%. The score was obtained by adding the intensity and reactivity. The scores < 2 represented low expression, the scores ≥ 2 represented the high expression.

### Cell migration and invasion assays

Cell migration and invasion assays were performed as previously described^24^. For the cell migration assays, an artificial “wound” was created after transfected SW480 cells (pIRES2-LIMK1 or pIRES2 empty vector) were cultured to 90% confluence. The cells were left untreated or treated with DADS (45 mg/L, Milwaukee, WI, USA) for 24 h, and the wound areas were then photographed using an inverted microscope at 0 h and 24 h. The migration distance was measured, and the migration rates were expressed as the ratio of the treated group value to the control group value. The invasion assays were performed using transwell plates (Corning, NY, USA). Briefly, SW480 cells were seeded onto Matrigel-coated filters. The transfected and untransfected cells were treated with indicated concentrations of DADS (45 mg/L) for 24 h or left untreated. Cells on the upper surface of filters were removed after 24 h, and those on the undersurface were fixed and stained with hematoxylin. Images were captured from each membrane, and the number of invasive cells was counted under a microscope, and the invasion rates were expressed as the ratio of the treated group value to the control group value.

### Cell proliferation assays and cell cycle analysis

The effect of DADS or LIMK1 on SW480 cell proliferation was measured using the 3-(4,5-dimethylthiazol-2-yl)-2,5-diphenyltetrazolium bromide (MTT) assay as previously described^24^. Briefly, 4 × 10^3^ cells were treated with or without DADS(45 mg/L) for 24 h. Subsequently, the cells were exposed to MTT(25 mL/well, 5 mg/mL) for 4 h. The formed formazan was dissolved in 0.15 mL of dimethyl sulfoxide, and the optical density values were measured at 490 nm.

The cell cycle analyses were conducted as follows^19^. Briefly, the cells were treated with 45 mg/L DADS for 24 h or left untreated. The cells were harvested and resuspended in ice-cold 75% ethanol and then fixed for 24 h at 4 °C. For the subsequent flow cytometry analysis, the fixed cells were resuspended in 1 mL of propidium iodide staining reagent for 30 min. The data were collected using a FACScan flow cytometer (Becton Dickinson, NJ, USA) and analyzed using Verity Winlist Software (Verity Software House, ME, USA).

### RT-PCR

A total RNA kit (Invitrogen, Breda, Netherlands) was used to extract total tissue RNA, according to the kit instruction. The cDNA was synthesized by the avian myeloblastosis virus−mediated reverse transcription. Each gene primer was synthesized by Shanghai Sangon. The sequences of primers were as follows: LIMK1 (length: 138 bp): forward: 5′-GGGGCATCATCAAGAGCA-3′, reverse: 5′-GAGGACTAGGGTGGTTCAG-3′; destrin (length: 276 bp): forward: 5′-TGGTTGGAGATGTTGGTG-3′, reverse: 5′-TACAAGCCCGATTGAGAT-3′; cofilin1 (length: 120 bp): forward: 5′-CAAGAAGGCGGTGCTCT3-3′, reverse: 5′-ACAAAGGTGGCGTAGGG-3′; β-actin (length: 367 bp): forward: 5′-ACACTGTGCCCATCTACGAGGGG-3′, reverse: 5′-ATGATGGAGTTGAAG GTAGTTTCGTGGAT-3′. PCR products were electrophoresed on 2% agarose gel (ethidium bromide staining), and the Bio-Rad gel imaging system (Bio-Rad Laboratories, Inc., CA, USA) was used to photograph. AlphaImager 2200 software (Alpha Innotech Corporation, San Leandro, CA, USA) was used to scan and calculate the relative optical density on behalf of the abundance of gene expression, and β-actin expression abundance was considered as a reference to calculate the mean optical density value.

### Western blot

SW480 cell lines were treated with or without DADS(45 mg/L), and the levels of targeting proteins were determined by western blot as described previously^25^, using primary antibodies, including anti-LIMK1, anti-destrin (Abcam, Cambridge, UK), anti-p-T508-LIMK1, anti-cofilin1, anti-p-cofilin1 (Abzoom Biolabs, Inc., TX, USA), and anti-GAPDH (Santa Cruz, CA, USA). The bound antibodies were detected using horseradish peroxidase–conjugated secondary antibodies and visualized using enhanced chemiluminescence (Pierce, IL, USA). The relative levels of individual proteins to control GAPDH were analyzed using ImageJ software (NIH, MD, USA).

### Assessment of *in vivo* tumor growth

The *in vivo* tumor growth was assessed as previously described^24^. The SW480 cells were injected into the subcutis of the right axilla of male athymic BALB/c nude mice (4 weeks old). The mice were intraperitoneally injected with normal saline or DADS(100 mg/kg) every 2 days until the termination of the experiment. The tumor volume (cm^3^) was examined every 6 days and calculated using a standard formula (width^2^ × length × 0.5). Average tumor volumes were presented (*n* = 5 for each group) starting from the 12th day and continuing until the mice were killed after 48 days. The xenografts were removed, and the tumor size and weight were measured after 48 days. The tumor tissues were then fixed in formalin and embedded in paraffin. The tissue sections (5 μm thick) were prepared for the subsequent immunohistochemical analysis. All experimental procedures were approved by the ethics committee of Animal Research at the University of South China (Hengyang, China) and conducted in accordance with the international guidelines for care and use of laboratory animals.

### Statistical analysis

All analyses were performed using the SPSS 15.0 program for Windows software package (SPSSInc., IL, USA). Statistical significance between groups within experiments was determined using the one-way analysis and Student *t* test. The chi-square test was used to determine whether two groups had distinct gene expression levels. The Spearman’s rank correlation test was used to determine the correlations between LIMK1 and destrin. The survival was estimated using the Kaplan–Meier method and compared using the log-rank test. A *P* value of < 0.05 was considered statistically significant.

## Results

### High expression of LIMK1 and destrin (also known as ADF) in colon cancer tissue

**Figure 1:**
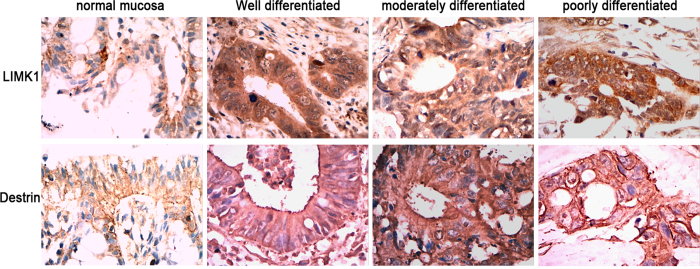
High expression of LIMK1 and destrin in colon cancer tissues. Representative images of LIMK1 and destrin, as detected by immunohistochemical analysis in normal mucosa and tumor samples (magnification x400). Brown denotes a positive signal.

The expression of LIMK1 and destrin was analyzed using the immunohistochemical staining of the colon cancer tissue array to evaluate the relationship between the expression of LIMK1 and destrin and colon cancer. The expression of LIMK1 and destrin was found to be low in the normal tissue of colonic mucosa (Fig. 1), with only 20.59% (7/34) cases with high expression. However, they were highly expressed in colon cancer tissue, with 75.86% (66/87) and 80.46% (70/87) cases with high expression, which were significantly higher compared with the normal tissue of colonic mucosa (*P  *<0.05). It indicated that the poorer the differentiation of colon cancer, the higher the expression of LIMK1 and destrin. The present results suggested that the significantly high expression of LIMK1 and destrin was related to the occurrence and development of colon cancer.

### Expression of LIMK1and destrin was closely related to the prognosis and clinicopathological parameters of colon cancer

**Figure 2:**
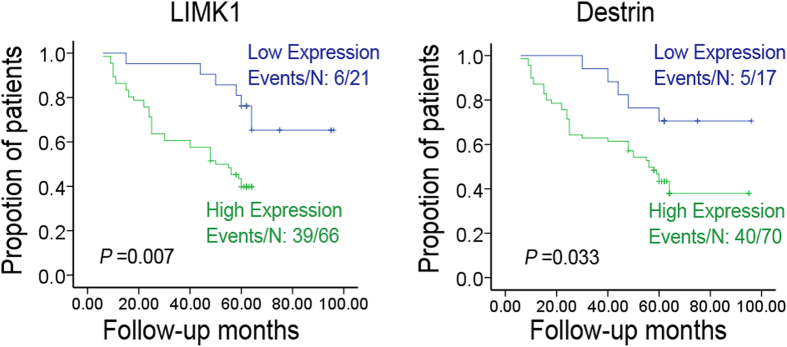
Expression of LIMK1 and destrin was significantly correlated with the survival of patients with colon cancer. Kaplan−Meier estimated OS for patients with colon cancer according to the expression of LIMK1 and destrin. The OS curves for patients with low or high expression of LIMK1 are shown. The increased expression of LIMK1 and destrin was correlated with poor OS. *P* values were obtained using the log-rank test. *N*, Number of cases; events, thenumber of patients who had arecurrent tumor or died during the follow-up period.

The relationship between the expression of LIMK1 and destrin and clinicopathological parameters and prognosis was analyzed to clarify the clinical significance of the expression of LIMK1 and destrin, and the role of prognostic monitoring in colon cancer. As shown in Table 1, the expression of LIMK1 and destrin was related to the pathological degree of differentiation (*P* = 0.001, *P* = 0.022), tumor size (*P* = 0.001, *P* = 0.004), clinical stage (*P* = 0.002, *P* = 0.007), and metastasis (*P* < 0.001, *P* = 0.009) in colon cancer, but not to gender and age. The Kaplan–Meier survival curve analysis showed that the expression of LIMK1 and destrin was closely associated with the overall survival (OS) of colon cancer (Fig. 2). Also, patients with colon cancer having a low expression of LIMK1 and destrin had a significantly longer OS compared with the patients having high expression [80.469 ± 5.629 vs 43.322 ± 2.665, hazard ratio (HR) = 3.084, *P* = 0.007; 80.824 ± 5.847 vs 56.335 ± 4.304,HR = 2.617, *P* = 0.033]. Moreover, the expression of LIMK1 was positively correlated with the expression of destrin (*r* = 0.487, *P* < 0.001) in colon cancer. The results further illustrated that the expression of LIMK1 and destrin promoted the development of colon cancer, and the high expression of LIMK1 and destrin was a potential molecular marker for poor prognostic monitoring in colon cancer.

### Overexpression of LIMK1 weakened the effect of DADS on colon cancer cell migration and invasion

**Figure 3:**
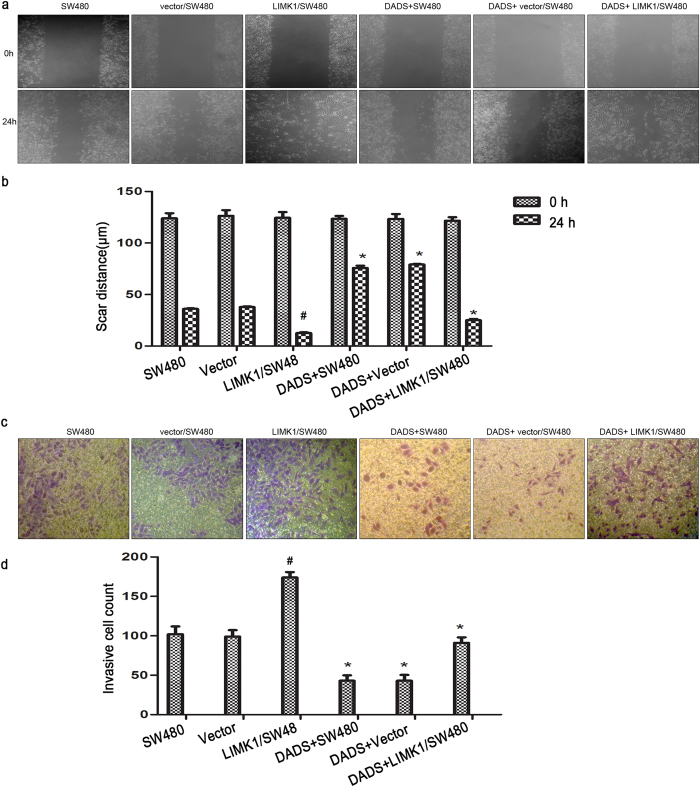
Overexpressionof LIMK1 weakened the effect of DADS on colon cancer cell migration and invasion. (**a**) Scratch wound-healing assay was used to detect the effect of DADS treatment and overexpressionof LIMK1 on SW480 cell migration. (**b**) Scar distance histograms of each group. (**c**) Invasion assay was used to detect the effect of DADS treatment and overexpression of LIMK1 on SW480 cell invasion. (**d**) Histograms showing the numbers penetrating cells in each group. *P < 0.05 vs SW480 or vector group; ^#^*P* < 0.05 vs SW480, vector, DADS + vector or DADS + SW480, and DADS + LIMK1/SW480 groups.

It was previously found that DADS could inhibit cancer cell migration and invasion. Also, silencing the expression of LIMK1 might enhance the inhibitory effect of DADS on colon cancer cell migration and invasion^25^. This study examined whether the overexpression of LIMK1 could antagonize the inhibitory effect of DADS on colon cancer cell migration and invasion to further define the role of *LIMK1* gene in the inhibitory effect of DADS on cancer cell migration and invasion. The scratch wound-healing assay showed that compared with the untreated and empty vector groups, the overexpression of LIMK1 in colon cancer cells led to faster wound healing, and cell migration was significantly enhanced. After DADS (45 mg/L) treatment, colon cancer cell migration could be significantly reduced, but the overexpression of LIMK1 could weaken such inhibition by DADS (Fig. 3a). The cell invasion assay also showed that DADS could significantly reduce the invasiveness of SW480 cells (*P* < 0.001), whereas the overexpression of LIMK1 significantly promoted SW480 cell invasion (*P* = 0.004), and might weaken the inhibitory effect of DADS on SW480 cell invasion (Fig. 3b). The results illustrated that the expression of LIMK1 promoted SW480 cell migration and invasion, and DADS inhibited SW480 cell migration and invasion due to the downregulation of LIMK1 expression.

### DADS inhibited the expression and phosphorylation of LIMK1 in colon cancer cells

**Figure 4:**
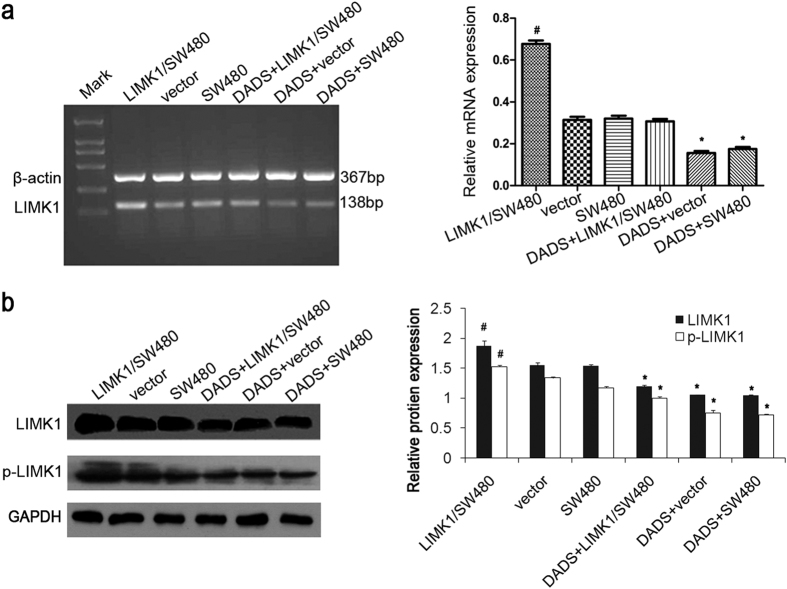
DADS inhibited the expression and phosphorylationof LIMK1 in colon cancer cells. (**a**) RT-PCR was used to detect the effect of DADS on the expression of LIMK1 mRNA in colon cancer cells with or without transfection. (**b**) Western blot was used to detect the effect of DADS on the expression level of LIMK1 protein (normalized to GAPDH) in colon cancer cells with or without transfection. *P < 0.05 vs SW480 or vector group; ^#^*P* < 0.05 vs SW480, vector, DADS + vector or DADS + SW480, and DADS + LIMK1/SW480 groups.

It was found previously that DADS could downregulate the expression and phosphorylation of LIMK1 in SW480 colon cancer cells. To further clarify the inhibitory effect of DADS on the expression of LIMK1, plasmids overexpressing exogenous LIMK1 were constructed to improve the expression of LIMK1 in colon cancer cells. Figure 4a,b show that the expression of LIMK1 mRNA and protein in the LIMK1 overexpression group was significantly upregulated compared with the empty vector and untreated groups; the level of p-LIMK1 protein also significantly increased (*P* < 0.05). However, after DADS treatment, the expression of LIMK1 mRNA and protein was significantly lower (*P* < 0.05) regardless of the exogenous overexpression of LIMK1 in the cells; the level of p-LIMK1 protein also significantly decreased (Fig. 4a,b). The results indicated that DADS could downregulate the expression of LIMK1 in SW480 cells and inhibit the phosphorylation level of LIMK1.

### DADS inhibited the expression of ADF/cofilin1 by downregulating the expression of LIMK1

**Figure 5:**
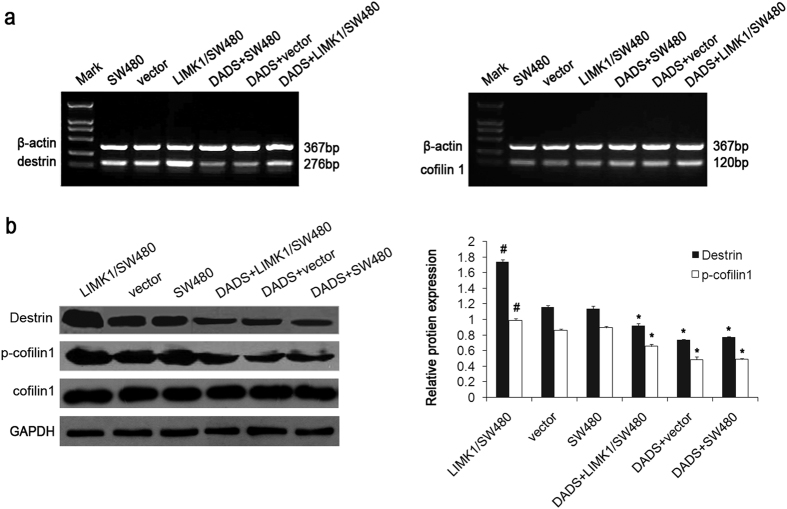
DADS inhibited the expression of destrin (ADF) and p-cofilin1 in colon cancer cells. **(a)** RT-PCR was used to detect the effect of DADS on the expression of destrin andcofilin1mRNA in colon cancer cells with or without transfection. **(b)** Western blot was used to detect the effect of DADS on the protein expression of destrin (normalized to GAPDH), cofilin1, and p-cofilin1 (normalized to cofilin1) in colon cancer cells with or without transfection.*P < 0.05 vs SW480 or vector group; ^#^*P* < 0.05 vs SW480, vector, DADS + vector or DADS + SW480, and DADS + LIMK1/SW480 groups.

It has been shown that activation mediated by the phosphorylation of LIMK1 can regulate the phosphorylation of ADF/cofilin to affect cytoskeleton remodeling, thereby regulating cell motility^26^. A previous study also showed that DADS could downregulate the expression of ADF/cofilin^23^, but it still needed to be further confirmed. The present study investigated whether DADS could downregulate LIMK1 in colon cancer cells with the overexpression of LIMK1, inhibiting the expression of ADF/cofilin. It was found that after DADS treatment in colon cancer cells, the expression of destrin mRNA and protein was significantly suppressed, while the overexpression of LIMK1 significantly upregulated the expression of destrin and weakened the inhibition of the expression of destrin by DADS (Fig. 5a,b). Although the overexpression of LIMK1 and DADS had no effect on the expression of total cofilin1 protein, DADS could inhibit the phosphorylation of cofilin1 protein in colon cancer cells, whereas the overexpression of LIMK1 significantly promoted the phosphorylation of cofilin1 protein and antagonized cofilin1 dephosphorylation by DADS. The results indicated that DADS could inhibit the expression of ADF/cofilin1 by downregulating the expression of LIMK1, thereby inhibiting the migration and invasion of SW480 cells.

### Effect of overexpression and silencing of LIMK1 ontheDADS-mediatedinhibition of colon cancer cell growth *in vitro* and *in vivo*

**Figure 6:**
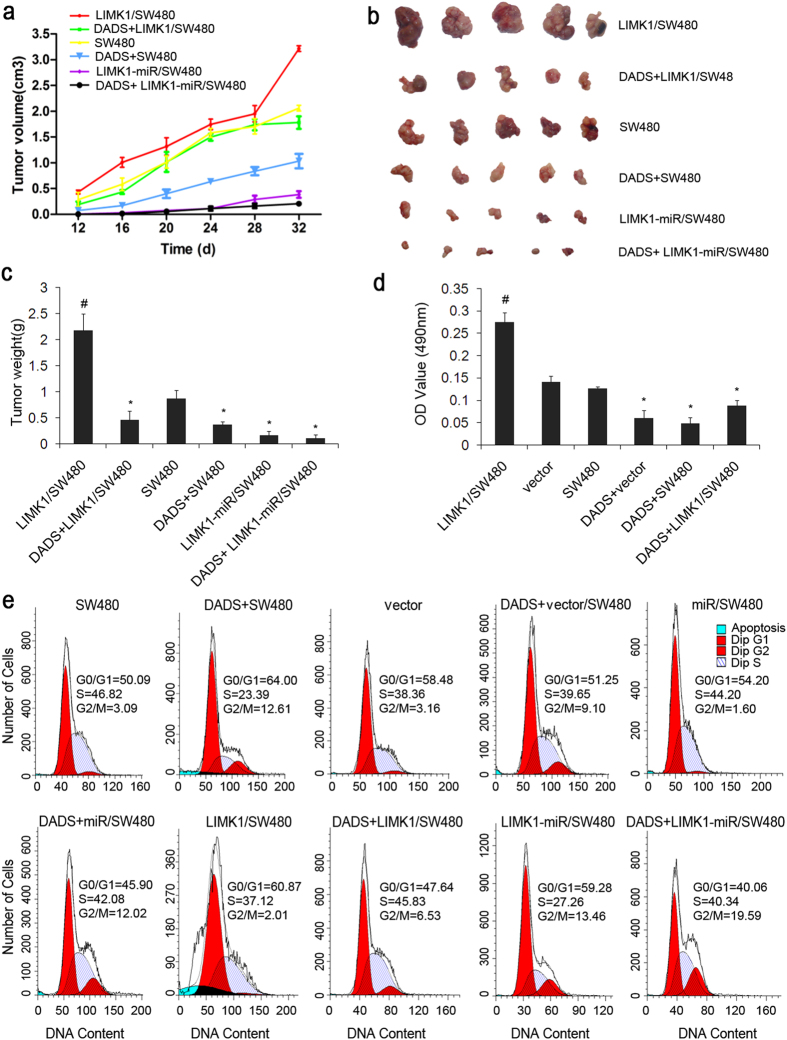
Effects of knockdown and overexpression of LIMK1 on the DADS-induced inhibition of growth in SW480 cells *in vitro* and *in vivo*. (**a**) SW480, LIMK1-miR-expressing SW480 cells, and LIMK1-overexpressing SW480 cells were injected into the subcutis of nude mice. The mice were intraperitoneally injected with normal saline or DADS every 2 days. The effect of DADS, LIMK1 knockdown, and overexpression of LIMK1 on tumor volume was examined every 6 days. Average tumor volumes were evaluated (*n* = 5 per group) starting from the 12th day and continuing until sacrifice at 48 days. (**b**) Xenograftswere collected after 48 days. Tumor sizes are shown. (**c**) Mean ± standard deviation tumor weight for each group was calculated at the termination of the experiment. (**d**) Cell proliferation assays were performed *in vitro* after the empty vector and LIMK1-overexpressing SW480 cells were treated with 45 mg/L DADS or left untreated for 24 h. (**e**) The cells were treated with 45 mg/L DADS or left untreated for 24 h. The percentages of cells in the G0/G1, S, and G2/M phases of the cell cycle were determined using flow cytometry. *P < 0.05 vs SW480 or vector group; ^#^*P* < 0.05 vs the other groups.

To evaluate the role of *LIMK1* gene in the inhibitory effect of DADS on colon cancer cell growth, the effect of changes in the expression of *LIMK1* gene on the inhibitory effect of DADS was analyzed *in vivo*. Experiments with xenografts in nude mice showed tumor formation in each group, which increased gradually with time. Comparison with the other groups indicated that tumor growth with the overexpression of LIMK1 was faster, but the tumor growth decreased significantly after DADS treatment. Compared with the empty vector and SW480 groups, tumor growth in LIMK1 silencing in DADS treatment groups was slower (*P* < 0.05). Silencing the expression of LIMK1 could enhance the inhibitory effect of DADS on tumor growth, whereas the overexpression of LIMK1 could weaken the inhibitory effect of DADS on tumor growth (Fig. 6a). All mice were sacrificed 45 days after inoculation, and tumors were isolated and weighed. The size, weight, and growth rate of tumors showed similar results (Fig. 6b,c). *In vitro* experiments demonstrated that the overexpression of LIMK1 significantly promoted the proliferation of colon cancer cells, and DADS significantly inhibited the proliferation of colon cancer cells and arrested the cell cycle in the G2/M phase (Fig. 6d,e). Thus, both *in vitro* and *in vivo* results showed that the upregulation of LIMK1 promoted colon cancer cell growth, and DADS could inhibit colon cancer growth by downregulating the expression of LIMK1.

### Effect of overexpression and silencing of LIMK1 ontheexpression of vimentin, E-cadherin, Ki-67, and CD34induced by DADS

**Figure 7:**
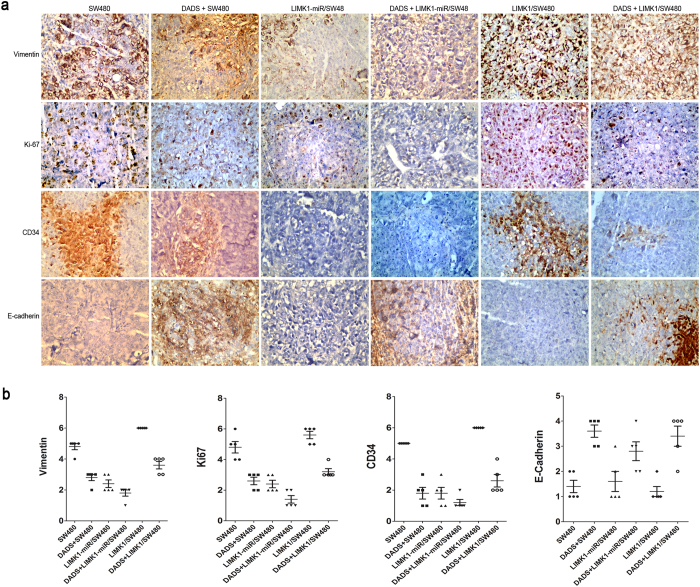
Effects of knockdown and overexpression of LIMK1 on the DADS-induced expression of vimentin, E-cadherin, CD34, and Ki-67. (**a**) Immunohistochemical analysis was performed to detect the expression of vimentin, CD34, Ki-67, and E-cadherin in the tumor tissue specimens obtained from the xenografts. A representative tissue section is shown for each group (x400 magnification). (**b**) Scatter plot showed the expression levels of vimentin, Ki-67, CD34, and E-cadherin of all five xenograftsfor each group.

Vimentin and CD34 are related to tumor growth, invasion, and metastasis; however, Ki67 protein is a marker of tumor cell proliferation. Therefore, immunohistochemical analysis was used to detect the expression of vimentin, Ki-67, and CD34 in colon cancer xenografts. As shown in Fig. 7a,b, the expression of vimentin, CD34, and Ki-67 was significantly lower in the DADS treatment and LIMK1 silencing groups. Silencing the expression of LIMK1 promoted the downregulation of the aforementioned molecules by DADS, and the overexpression of LIMK1 significantly increased the expression of vimentin, CD34, and Ki-67. The downregulation of E-cadherin might promote tumor cell invasion and metastasis. After DADS treatment or silencing the expression of LIMK1, the expression of E-cadherin significantly increased in colon cancer cells, whereas the overexpression of LIMK1 significantly reduced the expression of E-cadherin. Compared with DADS treatment alone, silencing the expression of LIMK1 enhanced the downregulation of the expression of E-cadherin by DADS, while the overexpression of LIMK1 antagonized it (Fig. 7a,b). The results suggested that DADS could downregulate the expression of LIMK1 to impair the expression of vimentin, CD34, and Ki-67 and increase the expression of E-cadherin, thereby inhibiting cancer growth, invasion, and metastasis.

## Discussion

The present study confirmed that LIMK1 and destrin (ADF) were overexpressed in primary colon cancer, which was correlated with the pathological grade, tumor size, clinical stage, lymph node metastasis, and poor prognosis of colon cancer. The expression of LIMK1 protein was positively correlated with the expression of destrin, suggesting that LIMK1 and destrin might contribute to carcinogenesis and the clinical progression of colon cancer. Subsequently, the study explored the effects of DADS on the LIMK1–ADF/cofilin signaling pathway, focusing on whether the downregulation of LIMK1 was related to the DADS-induced inhibition of the migration and invasion of colon cancer.

Numerous studies have confirmed that actin cytoskeleton remodeling is the basis for tumor cell migration, adhesion, and invasion^27^,^28^,^29^, and a variety of molecules are involved in the regulation of actin polymerization and depolymerization, of which the *LIMK* gene is an important molecule^30^,^31^. LIMK1 is overexpressed in a variety of malignancies including prostate, gastric, and lung cancers, and significantly promotes tumor cell invasion and metastasis^32^,^33^,^34^. Destrin is also expressed in all colon cancer cell lines examined, and involved in the colon cancer cell migration and invasion^35^. LIMK1 is involved in the migration and invasion of tumor cells in a number of ways, such as regulating the ADF/cofilin activity to affect actin cytoskeleton remodeling^36^, thereby promoting tumor cell migration and invasion^37^,^38^,^39^. In agreement with these reports, it was found that LIMK1 and its downstream molecule destrin (ADF) were highly expressed in colon cancer tissues. LIMK1 protein was positively correlated with the expression of destrin. Furthermore, the overexpression of LIMK1 or destrin was closely positively related to the pathological grade, tumor size, clinical stage, lymph node metastasis, and poor prognosis of colon cancer, while the overexpression of LIMK1 significantly promoted colon cancer cell migration and invasion *in vitro*. The results suggested that the overexpression of LIMK1 and destrin promoted the progression and metastasis of colon cancer. Hence, LIMK1 and destrin might serve as potential molecular markers for poor prognostic monitoring in colon cancer.

It is well known that tumor cell migration and invasion are key factors for the occurrence of distant metastasis. Thus, targeted inhibition of genes involved in tumor cell migration and invasion provides a new direction to anticancer therapy. DADS, a fat-soluble substance extracted from garlic, can decrease carcinogen-induced experimental cancers and inhibit the proliferation of various types of cancer cells. Its mechanisms of action include the suppression of DNA adduct formation, antioxidant activity, regulation of cell cycle arrest, induction of apoptosis and differentiation, histone modification, inhibition of angiogenesis and invasion, and so forth^40^. A previous study indicated that DADS had an inhibitory effecton various cancers, such as gastric cancer, colon cancer, and leukemia^24^,^41^,^42^,^43^,^44^,^45^. DADS could significantly decrease the expression of LIMK1 and destrin in SW480 human colon cancer cells. The reduction in the expression of LIMK1 had a synergistic effect with DADS treatment for inhibiting human colon cancer cell invasion and migration^25^. A colon cancer cell line with the stable expression of LIMK1was established in the present study to further clarify the role of LIMK1 in DADS-mediated inhibitory effect on colon cancer cell proliferation, migration, and invasion. *In vivo* and *in vitro* experiments showed that DADS did suppress colon cancer cell proliferation, invasion, and migration with the low expression of LIMK1 and p-LIMK1, while the exogenously elevated expression of LIMK1 might weaken the DADS-mediated inhibitory effect on colon cancer cell migration and invasion, consistent with previous findings that LIMK1 promoted tumor invasion and metastasis. LIMK inhibitors or knocking down the expression of LIMK1 significantly inhibited cancer cell invasion and metastasis^32^,^33^,^34^,^46^,^47^. The results showed that the upregulation of LIMK1 promoted colon cancer cell proliferation, invasion, and migration. Also, DADS could inhibit colon cancer growth, invasion, and migration by downregulating the expression of LIMK1. Hence, LIMK1 might be a potential target of DADS.

The mechanism by which LIMK1 promotes tumor cell invasion and metastasis is complex^5^. Actin is a major component of pseudopodia and also a major player resulting in tumor cell invasion and metastasis^48^. The overexpression/activation of LIMK1 promotes the phosphorylation of downstream signaling molecules ADF/cofilin. It reduces the activation of depolymerization of ADF/cofilin. This subsequently interferes with actin depolymerization, promotes actin cytoskeletal remodeling and cell pseudopodia formation, and induces EMT, thereby promoting tumor invasion and metastasis^36^,^49^. The present study further confirmed that the expression and phosphorylation levels of LIMK1 significantly increased during colon cancer cell invasion and migration. However, DADS significantly downregulated the expression of LIMK1 and inhibited the phosphorylation of LIMK1, accompanied by the downregulation of destrin mRNA and protein and inhibition of phosphorylation of cofilin1, which were downstream genes of LIMK1. These findings showed that DADS could downregulate the expression of LIMK1 to inhibit the expression of ADF/cofilin1, thereby inhibiting the migration and invasion of SW480 cells.

It is also known that tumor cells with a high rate of proliferation, angiogenesis, and EMT alteration are prone to metastasis^50^. Ki67 and CD34 are markers of tumor cell proliferation and angiogenesis, respectively^51^. Vimentin and E-cadherin are molecular markers of EMT. The cells with the high expression of vimentin and E-cadherin suggest that tumor cells are prone to EMT, hence promoting cell invasion and metastasis^52^. The present study found that the overexpression of LIMK1 upregulated the expression of vimentin, CD34, and Ki-67, and decreased the expression of E-cadherin. DADS treatment or silencing the expression of LIMK1 could significantly reverse the aforementioned changes, and silencing the expression of LIMK1 had a synergistic effect with DADS treatment. The results suggested that DADS downregulated LIMK1 expression to decrease the expression of vimentin, CD34, and Ki-67; increase E-cadherin expression; and impede cancer cell proliferation, angiogenesis, and EMT alteration, thereby inhibiting invasion and metastasis. However, this needs to be further confirmed.

In summary, the present study showed that LIMK1 and destrin (ADF) were highly expressed in colon cancer tissue, and the overexpression of LIMK1 significantly promotedcolon cancer cell migration and invasion. DADS can downregulate the expression of LIMK1 to inhibit the LIMK1–ADF/cofilin signaling pathway, and impede angiogenesis and EMT, thereby inhibiting the migration and invasion of colon cancer. The study further demonstrated that DADS had a significant anticancer effect, and LIMK1 was a potential target molecule of DADS for inhibiting tumor cell migration and invasion. It provided experimental evidence for using DADS as an anticancer drug in the clinic and a targeted molecule for the therapy of tumor invasion and metastasis.

## Additional Information

**How to cite this article:** Su, J. *et al*. Downregulation of LIMK1-ADF/cofilin by DADS inhibits the migration and invasion of colon cancer. *Sci. Rep.* **7**, 45624; doi: 10.1038/srep45624 (2017).

**Publisher's note:** Springer Nature remains neutral with regard to jurisdictional claims in published maps and institutional affiliations.

## Figures and Tables

**Table 1:** Relationships between the expression of LIMK1 and destrin and clinicopathological characteristics.

Variable	*N*	LIMK1			Destrin		
		High	Low	*P* Value	High	Low	*P* Value
Age (year)							
<50	38	28	10	0.676	30	8	0.754
≥50	49	38	11		40	9	
Gender							
Male	58	43	15	0.595	46	12	0.702
Female	29	23	6		24	5	
Tumor size (cm)							
< 3	35	20	15	0.001	23	12	0.004
≥3	52	46	6		47	5	
Histological grading							
Well differentiation	10	4	6	0.001	5	5	0.022
Moderatedifferentiation	47	33	14		38	9	
Poordifferentiation	30	29	1		27	3	
TNM stages							
I + II	41	25	16	0.002	28	13	0.007
III + IV	46	41	5		42	4	
Lymph node metastasis							
Yes	45	44	1	0.000	41	4	0.009
No	42	22	20		29	13	

## References

[b1] Siegel R. L., Miller K. D. & Jemal A. Cancer statistics, 2015. CA Cancer J Clin. 65, 5–29 (2015). 25559415 10.3322/caac.21254

[b2] Paz H., Pathak N. & Yang J. Invading one step at a time: the role of invadopodia in tumor metastasis. Oncogene. 33, 4193–202 (2014). 24077283 10.1038/onc.2013.393 PMC3969876

[b3] Saykali B. A. & El-Sibai M. Invadopodia, regulation, and assembly in cancer cell invasion. Cell CommunAdhes. 21, 207–12 (2014). 10.3109/15419061.2014.923845 24930891

[b4] Revach O. Y., Winograd-Katz S. E., Samuels Y. & Geiger B. The involvement of mutant Rac1 in the formation of invadopodia in cultured melanoma cells. Exp Cell Res. 343, 82–8 (2016). 26873115 10.1016/j.yexcr.2016.02.003 PMC4954600

[b5] Lagoutte E. . LIMK Regulates Tumor-Cell Invasion and Matrix Degradation Through Tyrosine Phosphorylation of MT1-MMP. Sci Rep. 6, 24925 (2016). 27116935 10.1038/srep24925 PMC4847008

[b6] Manetti F. LIM kinases are attractive targets with many macromolecular partners and only a few small molecule regulators. Med Res Rev. 32, 968–98 (2012). 22886629 10.1002/med.20230

[b7] Bernard O. Lim kinases, regulators of actin dynamics. Int J Biochem Cell Biol. 39, 1071–6 (2007). 17188549 10.1016/j.biocel.2006.11.011

[b8] Cai S. . Overexpression of P21-activated kinase 4 is associated with poor prognosis in non-small cell lung cancer and promotes migration and invasion. J ExpClin Cancer Res. 34, 48 (2015). 10.1186/s13046-015-0165-2 PMC4443662 25975262

[b9] Hamill S., Lou H. J., Turk B. E. & Boggon T. J. Structural Basis for Noncanonical Substrate Recognition of Cofilin/ADF Proteins by LIM Kinases. Mol Cell. 62, 397–408 (2016). 27153537 10.1016/j.molcel.2016.04.001 PMC4860616

[b10] Manetti F. Recent findings confirm LIM domain kinases as emerging target candidates for cancer therapy. Curr Cancer Drug Targets. 12, 543–60 (2012). 22414009 10.2174/156800912800673266

[b11] urhani D., Krapfenbauer K., Thurnher D., Langen H. & Fountoulakis M. Identification of differentially expressed, tumor-associated proteins in oral squamous cell carcinoma by proteomic analysis. Electrophoresis. 27, 1417–23 (2006). 16568407 10.1002/elps.200500510

[b12] Maimaiti Y. . Dephosphorylated cofilin expression is associated with poor prognosis in cases of human breast cancer: a tissue microarray analysis. Onco Targets Ther. 9, 6461–6466 (2016). 27799793 10.2147/OTT.S107321 PMC5077260

[b13] Wang Y. . Cofilin-phosphatase slingshot-1L (SSH1L) is over-expressed in pancreatic cancer (PC) and contributes to tumor cell migration. Cancer Lett. 360, 171–6 (2015). 25684665 10.1016/j.canlet.2015.02.015

[b14] Wang W. . Identification and testing of a gene expression signature of invasive carcinoma cells within primary mammary tumors. Cancer Res. 64, 8585–94 (2004). 15574765 10.1158/0008-5472.CAN-04-1136

[b15] Wang W., Eddy R. & Condeelis J. The cofilin pathway in breast cancer invasion and metastasis. Nat Rev Cancer. 7, 429–40 (2007). 17522712 10.1038/nrc2148 PMC4270061

[b16] Lourenco F. C. . Reduced LIMK2 expression in colorectal cancer reflects its role in limiting stem cell proliferation. Gut. 63, 480–93 (2014). 23585469 10.1136/gutjnl-2012-303883 PMC3932979

[b17] Ding Y., Milosavljevic T. & Alahari S. K. Nischarin inhibits LIM kinase to regulate cofilin phosphorylation and cell invasion. Mol Cell Biol, 28, 3742–56 (2008). 18332102 10.1128/MCB.01832-07 PMC2423293

[b18] Saud S. M. . Diallyl Disulfide (DADS), a constituent of garlic, inactivates NFkappaB and prevents colitis-induced colorectal cancer by inhibiting GSK-3beta. Cancer Prev Res (Phila)(2016). 10.1158/1940-6207.CAPR-16-0044 PMC4930730 27138790

[b19] Manral A., Saini V., Meena P. & Tiwari M. Multifunctional novel Diallyl disulfide (DADS) derivatives with beta-amyloid-reducing, cholinergic, antioxidant and metal chelating properties for the treatment of Alzheimer’s disease. Bioorg Med Chem. 23, 6389–403 (2015). 26337018 10.1016/j.bmc.2015.08.024

[b20] Su B. . Identification of potential targets for diallyl disulfide in human gastric cancer MGC-803 cells using proteomics approaches. Oncol Rep. 33, 2484–94 (2015). 25812569 10.3892/or.2015.3859

[b21] Huang J. . Diallyl disulfide inhibits growth and metastatic potential of human triple-negative breast cancer cells through inactivation of the beta-catenin signaling pathway. MolNutr Food Res 59, 1063–75 (2015). 10.1002/mnfr.201400668 25755089

[b22] Suangtamai T. &Tanyong D. I. Diallyl disulfide induces apoptosis and autophagy via mTOR pathway in myeloid leukemic cell line. Tumour Biol(2016). 10.1007/s13277-016-4989-y 26891668

[b23] Su J. . The differential proteomic expression analysis of diallyl disulfide-induced human colonic cancer cells. Chinese Pharmacological Bulletin. 22, 5 (2006).

[b24] Su B. . Diallyl disulfide suppresses epithelial-mesenchymal transition, invasion and proliferation by downregulation of LIMK1 in gastric cancer. Oncotarget. 7, 10498–512 (2016). 26871290 10.18632/oncotarget.7252 PMC4891135

[b25] Zhou Y., Su J., Shi L., Liao Q. & Su Q. DADS downregulates the Rac1-ROCK1/PAK1-LIMK1-ADF/cofilin signaling pathway, inhibiting cell migration and invasion. Oncol Rep. 29, 605–12 (2013). 23233092 10.3892/or.2012.2168

[b26] Van Troys M. . Ins and outs of ADF/cofilin activity and regulation. Eur J Cell Biol. 87, 649–67 (2008). 18499298 10.1016/j.ejcb.2008.04.001

[b27] Flamini M. I. . Effects of raloxifene on breast cancer cell migration and invasion through the actin cytoskeleton. J Cell Mol Med. 13, 2396–407 (2009). 18798864 10.1111/j.1582-4934.2008.00505.x PMC6512380

[b28] Freitas V. M., Rangel M., Bisson L. F., Jaeger R. G. & Machado-Santelli G. M. The geodiamolide H, derived from Brazilian sponge Geodiacorticostylifera, regulates actin cytoskeleton, migration and invasion of breast cancer cells cultured in three-dimensional environment. J Cell Physiol. 216, 583–94 (2008). 18330887 10.1002/jcp.21432

[b29] Yamaguchi H. & Condeelis J. Regulation of the actin cytoskeleton in cancer cell migration and invasion. BiochimBiophysActa. 1773, 642–52 (2007). 10.1016/j.bbamcr.2006.07.001 PMC4266238 16926057

[b30] Ishaq M. . LIM kinase 1 - dependent cofilin 1 pathway and actin dynamics mediate nuclear retinoid receptor function in T lymphocytes. BMC Mol Biol. 12, 41 (2011). 21923909 10.1186/1471-2199-12-41 PMC3187726

[b31] Nadella K. S. . Regulation of actin function by protein kinase A-mediated phosphorylation of Limk1. EMBO Rep. 10, 599–605 (2009). 19424295 10.1038/embor.2009.58 PMC2711837

[b32] Cai S. . Downregulation of microRNA-23a suppresses prostate cancer metastasis by targeting the PAK6-LIMK1 signaling pathway. Oncotarget. 6, 3904–17 (2015). 25714010 10.18632/oncotarget.2880 PMC4414162

[b33] You T. . Overexpression of LIMK1 promotes tumor growth and metastasis in gastric cancer. Biomed Pharmacother. 69, 96–101 (2015). 25661344 10.1016/j.biopha.2014.11.011

[b34] Wan L., Zhang L., Fan K. & Wang J. MiR-27b targets LIMK1 to inhibit growth and invasion of NSCLC cells. Mol Cell Biochem. 390, 85–91 (2014). 24390089 10.1007/s11010-013-1959-1

[b35] Estornes Y. . Differential involvement of destrin and cofilin-1 in the control of invasive properties of Isreco1 human colon cancer cells. Int J Cancer. 121, 2162–71 (2007). 17583572 10.1002/ijc.22911

[b36] Liu A. . Neuroligin 1 regulates spines and synaptic plasticity via LIMK1/cofilin-mediated actin reorganization. J Cell Biol. 212, 449–63 (2016). 26880202 10.1083/jcb.201509023 PMC4754719

[b37] Konakahara S., Ohashi K., Mizuno K., Itoh K. & Tsuji T. CD29 integrin- and LIMK1/cofilin-mediated actin reorganization regulates the migration of haematopoietic progenitor cells underneath bone marrow stromal cells. Genes Cells. 9, 345–58 (2004). 15066125 10.1111/j.1356-9597.2004.00726.x

[b38] Borensztajn K., Peppelenbosch M. P. & Spek C. A. Coagulation Factor Xa inhibits cancer cell migration via LIMK1-mediated cofilin inactivation. Thromb Res. 125, e323–8 (2010). 20347121 10.1016/j.thromres.2010.02.018

[b39] Chen P., Zeng M., Zhao Y. & Fang X. Upregulation of Limk1 caused by microRNA-138 loss aggravates the metastasis of ovarian cancer by activation of Limk1/cofilin signaling. Oncol Rep. 32, 2070–6 (2014). 25190487 10.3892/or.2014.3461

[b40] Yi L. & Su Q. Molecular mechanisms for the anti-cancer effects of diallyl disulfide. Food Chem Toxicol, 57, 362–70 (2013). 23583486 10.1016/j.fct.2013.04.001

[b41] Bo S. . Chk1, but not Chk2, is responsible for G2/M phase arrest induced by diallyl disulfide in human gastric cancer BGC823 cells. Food ChemToxicol. 68, 61–70 (2014). 10.1016/j.fct.2014.03.007 24650757

[b42] Liao Q. J. . Effect of diallyl disulfide on cell cycle arrest of human colon cancer sw480 cells. Ai Zheng. 28, 168–72 (2009). 19550124

[b43] Huang Y. S. . Diallyl disulfide inhibits the proliferation of HT-29 human colon cancer cells by inducing differentially expressed genes. Mol Med Rep. 4, 553–9 (2011). 21468607 10.3892/mmr.2011.453

[b44] Yi L. . Involvement of Mcl1 in diallyl disulfide-induced G2/M cell cycle arrest in HL-60 cells. Oncol Rep. 27, 1911–7 (2012). 22378300 10.3892/or.2012.1704

[b45] Zhao J. . Diallyl disulfide suppresses growth of HL-60 cell through increasing histone acetylation and p21^WAF1^ expression *in vivo* and *in vitro*. Acta Pharmacol Sin. 27, 1459–66 (2006). 17049122 10.1111/j.1745-7254.2006.00433.x

[b46] Kang C. G., Lee H. J., Kim S. H. & Lee E. O. Zerumbone Suppresses Osteopontin-Induced Cell Invasion Through Inhibiting the FAK/AKT/ROCK Pathway in Human Non-Small Cell Lung Cancer A549 Cells. J Nat Prod. 79, 156–60 (2016). 26681550 10.1021/acs.jnatprod.5b00796

[b47] Li H., Zhang B., Liu Y. & Yin C. EBP50 inhibits the migration and invasion of human breast cancer cells via LIMK/cofilin and the PI3K/Akt/mTOR/MMP signaling pathway. Med Oncol. 31, 162 (2014). 25119502 10.1007/s12032-014-0162-x

[b48] Bisi S. . Membrane and actin dynamics interplay at lamellipodia leading edge. CurrOpin Cell Biol. 25, 565–73 (2013). 10.1016/j.ceb.2013.04.001 23639310

[b49] Hong K. O., Lee J. I., Hong S. P. & Hong S. D. Thymosin beta4 induces proliferation, invasion, and epithelial-to-mesenchymal transition of oral squamous cell carcinoma. Amino Acids. 48, 117–27 (2016). 26276576 10.1007/s00726-015-2070-6

[b50] Guan X. Cancer metastases: challenges and opportunities. Acta Pharm Sin B. 5, 402–18 (2015). 26579471 10.1016/j.apsb.2015.07.005 PMC4629446

[b51] Ma Y. L. . Immunohistochemical analysis revealed CD34 and Ki67 protein expression as significant prognostic factors in colorectal cancer. Med Oncol. 27, 304–9 (2010). 19340613 10.1007/s12032-009-9210-3

[b52] Smith B. N. & Bhowmick N. A. Role of EMT in Metastasis and Therapy Resistance. J Clin Med. 5 (2016). 10.3390/jcm5020017 PMC4773773 26828526

